# Valorization of agro-industrial waste from the cassava industry as esterified cellulose butyrate for polyhydroxybutyrate-based biocomposites

**DOI:** 10.1371/journal.pone.0292051

**Published:** 2023-11-22

**Authors:** Passaraporn Theeraseematham, Duangdao Aht-Ong, Kohsuke Honda, Suchada Chanprateep Napathorn

**Affiliations:** 1 Department of Microbiology, Faculty of Science, Chulalongkorn University, Patumwan, Bangkok, Thailand; 2 Department of Materials Science, Faculty of Science, Chulalongkorn University, Patumwan, Bangkok, Thailand; 3 Center of Excellence on Petrochemical and Materials Technology, Chulalongkorn University, Bangkok, Thailand; 4 International Center for Biotechnology, Osaka University, Suita, Osaka, Japan; Universidad Tecnica de Ambato, ECUADOR

## Abstract

The aim of this study was to utilize cassava pulp to prepare biocomposites comprising microcrystalline cellulose from cassava pulp (CP-MCC) as a filler and polyhydroxybutyrate (PHB) synthesized in-house by *Cupriavidus necator* strain A-04. The CP-MCC was extracted from fresh cassava pulp. Next, the CP-MCC surface was modified with butyryl chloride (esterified to CP-MCC butyrate) to improve dissolution and compatibility with the PHB. FTIR results confirmed that the esterified CP-MCC butyrate had aliphatic chains replacing the hydroxyl groups; this substitution increased the solubilities in acetone, chloroform, and tetrahydrofuran. Biocomposite films were prepared by varying the composition of esterified CP-MCC butyrate as a filler in the PHB matrix at 0, 5, 10, 15, 20 and 100 wt%. The results for the 95:5 and 90:10 CP-MCC butyrate biocomposite films showed that esterification led to improvements in the thermal properties and increased tensile strengths and elongations at break. All prepared biocomposite films maintained full biodegradability.

## Introduction

Large volumes of agro-industrial wastes are generated along with food processing and are abundant renewable resources that require proper waste management. Meanwhile, practical waste management approaches must be based on a balance between economic and environmental benefits. In addition, the balance between increasing food demand and overall minimal impact on the environment should move toward sustainable development goals (SDGs) aimed at reducing food loss and waste along the food production chain by 2030 [1].

Economic interest is predicated on the possibility of producing new compounds with added value from a significant amount of these wastes, which would result in lower production costs. The environmental problem stems from their composition, particularly agro-industrial wastes that may include potentially harmful substances and may worsen the environment if they are burned, allowed to decompose naturally in the soil, or buried underground [2]. Furthermore, if not properly disposed of, these materials, which have high biological oxygen demand (BOD) and chemical oxygen demand (COD), could cause major pollution issues. If they are recycled and transformed into value-added products through a fermentation process, these food wastes present substantial potential to promote sustainable development [3]. Recently, the concept of zero-waste management has been adopted in food processing factories. Accordingly, the valorization of food waste by recycling and transformation into value-added products has provided an excellent opportunity to promote sustainable development.

Recently, one of the most urgent environmental concerns seems to be microplastics contamination [4]. Researchers are working quickly to understand these minute plastic particles found in the human body and marine animals [5]. With studies estimating a twofold increase in plastic litter (including micro- and nanosized plastics) by 2030, plastics have evolved into a serious transboundary threat to natural ecosystems and human health [6]. Due to the COVID-19 pandemic, there will certainly be an increase in the use and consumption of single-use plastics, including personal protective equipment such as masks and gloves. To solve the environmental problems arising from plastics pollution, many countries have prioritized reductions in plastic consumption and encouraged the use of biodegradable plastics. Both governments and private organizations have adopted policy measures consisting of two actions: taxation of plastic bags and legislation to ban plastic bags [7]. In addition, the pollution caused by waste synthetic plastics has driven research on alternatives. Bioplastics, which are environmentally friendly, are being promoted as potentially profitable materials with which to tackle these issues [8]. Based on biodegradation and raw materials, bioplastics can be divided into three groups: (1) biodegradable and fossil-based bioplastics such as polycaprolactone (PCL), polybutylene succinate (PBS), polybutylene adipate-co-terephthalate (PBAT) and polyvinyl alcohol (PVA); (2) nonbiodegradable and biobased bioplastics such as biopolyethylene (bio-PE), biopolyethylene terephthalate (bio-PET), and biopolypropylene (bio-PP); and (3) biodegradable and biobased plastics such as polylactic acid (PLA) and polyhydroxyalkanoates (PHAs) [9, 10]. As mentioned above, biocomposites made of microcrystalline cellulose from cassava pulp (CP-MCC) as a filler and polyhydroxybutyrate (PHB) synthesized in-house by *Cupriavidus necator* strain A-04 can be considered food waste valorizers.

Polyhydroxyalkanoates (PHAs) are bioplastics that have attracted interest in recent studies since they are produced biosynthetically by microorganisms and accumulate as intracellular granules used for carbon and energy storage in the cytoplasm. PHAs exhibit biodegradability, thermoplasticity, compatibility and generation from renewable carbon sources [11]. The number of atoms in the monomer is used to determine the type of PHA [10]. PHAs can be divided into two categories based on the number of carbon atoms in a monomer: (1) short-chain-length PHAs (scl-PHAs) consisting of 3–5 carbon atoms are found in *Cupriavidus necator*. The properties of scl-PHAs include high crystallinity (55–80%), high melting temperatures (173–180°C) and low glass transition temperatures (5–9°C), which result in stiffness and brittleness [12]; and (2) medium chain length PHAs (mcl-PHAs) contain 6–14 carbon atoms and are found in *Pseudomonas* sp. Mcl-PHAs have low crystallinities (25%), low melting temperatures (39–61°C) and low glass transition temperatures (-43–25°C), which result in greater flexibility than that of scl-PHAs [13]. Among the various types of PHAs, polyhydroxybutyrate (PHB) has been studied extensively because its physical properties are similar to those of polypropylene (PP), which is a synthetic plastic. However, PHB is hard and brittle, and more expensive than synthetic plastics. As a result, these disadvantages have limited its use [14]. Conversion of PHB into biocomposites with other polymers is one way to enhance its their thermal and mechanical features [15]. Due to the relatively high costs associated with PHA production and downstream processing, blending is one of the simplest ways to obtain biocomposite materials with improved functionalities and reduce the overall cost [16]. This can be done by choosing suitable and compatible natural polymers [17]. Generally, biocomposites consist of two or more materials with different chemical compositions and include a reinforcing agent or a filler distributed in the matrix. The matrix supports the filler material, which maintains the mechanical properties of the matrix. The use of cellulose as a filler material has advantages because cellulose is biodegradable, renewable, and economical [18]. Cellulose is the most abundant biopolymer synthesized by microorganisms, plants and animals, and it has good properties: it is biodegradable, nontoxic and renewable. However, cellulose exhibits high crystallinity ranging from 40 to 70%, depending on the kind of cellulose and the production process [19]. As a result, it is hydrophilic and does not dissolve in common organic solvents, which limits the solubility of the polymer in solvent blends containing organic solvents. Therefore, the chemical structure of cellulose must be modified to make it soluble in organic solvents and/or reduce its melting temperature. Cellulose modification by fatty acid esterification has been studied extensively and applied in various industries, such as foods, textiles and films [20].

In this study, cassava pulp generated from the cassava starch industry has attracted our interest in converting this abundant agro-industrial waste to microcrystalline cellulose (MCC) to be used as a filler for the PHB matrix. Cassava (*Manihot esculenta* Crantz) is one of the oldest root and tuber crops and has been grown by humans for food, feed, and drinks in over 100 nations [21]. In the primary producing countries, cassava is currently becoming a cash crop due to government promotion of cassava marketing, the establishment of the starch industry and the use of cassava as a raw material for the production of starch, including starch-based products, energy (bioethanol), and livestock feed [22]. Cassava became the 5^th^ most important crop in the world, after wheat, corn, rice and potatoes [23]. In 2021–2022, approximately 302 million tons of cassava were produced globally. The world’s largest cassava starch producer is Nigeria (21.6%), followed by Thailand (10.7%), with a 35.1 million ton annual production capacity [23, 24]. As a result, cassava pulp is also produced in significant quantities, and it accounts for approximately 10% of the raw material and comprises 40–60% of the residual starch bound to residual fibers [25]. Thus, cassava pulp is a readily available, affordable, abundant, and renewable form of agricultural waste [26]. Moreover, because cassava pulp is a nonedible crop and biomass waste, it is considered a second-generation biorefinery product [27]. Even though a large amount of cassava pulp is produced, a problem still exists because the process is poorly controlled and the cassava trash is often kept in an open area. As a result, it starts to spoil right away and gives off an offensive odor and pollutes ground water. According to previous reports, cassava pulp contains 40%–80% starch and 20%–40% fibers depending on the starch milling technology, plant variety and other environmental factors [28–30]. Sinsukudomchai et al studied a green composite made of PHB and long-chain fatty acid esterified microcrystalline cellulose from pineapple leaf (esterified PALF-MCC laurate). They reported that adding esterified PALF-MCC laurate as a filler in the biocomposite film could retain a pleasant value tensile strength and elastic modulus, whereas a slight increase in elongation enhanced the flexibility [31].

The purpose of this work is to study the effect of short-chain fatty acid esterified MCC from cassava pulp on the physical characteristics and biodegradability of biocomposite PHB/CP-MCC butyrate films. Accordingly, the cellulose extracted from food wastes could promote sustainability, support the SDG policy, and be considered for the valorization of agro-industrial food wastes.

## Materials and methods

### Materials

PHB was produced by *C*. *necator* strain A-04 in a 10-liter bioreactor (MDL-10 L, B.E. Marubishi Co., Ltd., Tokyo, Japan) [16, 17]. The C/N ratio was set to 200. The bioreactor was operated at 30°C for 60 h with an agitation speed of 500 rpm and an air flow rate of 1.88 vvm. The washed cells were dried at 65°C for 24 h. Filter paper (Whatman 1002–042, Sigma–Aldrich Corp., St. Louis, MO, USA) was used to extract the dried cells, which were then refluxed in hot chloroform in a Soxhlet apparatus. Then, PHB was recovered by precipitation with three volumes of n-hexane [32, 33].

Cassava bagasse (Manihot esculenta (L.) Crantz) used in the extraction of microcrystalline cellulose (MCC) was obtained from Thai Wah Public Company Limited (Tungmahamek, Sathorn, Bangkok, Thailand) and is a residual cassava left from the cassava starch production process. The cassava bagasse was dried overnight at 65°C in a hot-air oven (UN55, Memmert Co., Ltd., 136 Schwabach, Germany) and analyzed to determine its chemical composition. It was then milled in a laboratory blender (45,000 rpm, 1800 W, 137 Healthy mix GP 3.5, Taiwan). Different cassava bagasse particle sizes were separated by sieving with mesh sizes of 0.420 to 0.250 mm (40/+ 60 mesh). The chemical compositions of 100 grams of each size fraction, as well as their solubilities in alcohol and benzene, were investigated using TAPPI standard methods for the following parameters: benzene extractives (TAPPI T204 cm-07); α-cellulose, β-cellulose, and γ-cellulose (TAPPI 141 T203 om-09); holocellulose (TAPPI T9 m-54); lignin (TAPPI T222 om-15); and ash (TAPPI T-211).

### Analytical methods

Gel permeation chromatography (GPC; Shimadzu 10A GPC system, Shimadzu Co., Ltd., Kyoto, Japan) with a 10A refractive index detector and two Shodex columns (a GPC K-806 M column (8.0 mm ID 300 mm L, 165 Showa Denko K.K., Tokyo, Japan)) was used to determine the weight-average molecular weight (M_W_) and number-average molecular weight (M_N_), as well as the polydispersity index (PDI) from the ratio of $\frac{M_{W}}{M_{N}}$. PHB was dissolved in 0.1% (w/v) chloroform and filtered through a 0.45-m Durapore^®^ (PVDF) membrane filter (Millex^®^-HV, Merck Millipore Ltd., Tullagreen, Carrigtwohill Co., Cork, Ireland). The temperature and flow rate were set to 40°C and 0.8 mL/min, respectively. Under the same conditions, a standard curve was determined for polystyrenes with low polydispersity indices and molecular weights of 1.26×10^3^, 3.39×10^3^, 1.30×10^4^, 5.22×10^4^, 2.19×10^5^, 7.29×10^5^, 2.33×10^6^, and 7.45×10^6^. Chemical structural analyses of the PHB_A-04_ samples were performed with ^1^H NMR spectrometry with a Varian Inova 500 MHz instrument (Varian Inc., California, USA). The chemical shifts are reported in parts per million (ppm) relative to chloroform as an internal reference. Spectra were recorded with 5% (w/w) polymer solutions in CDCl_3_ with the following parameters: 25°C, 90°, pulse width of 8003.2 Hz, 5.0 s relaxation delay, and 0.2 Hz line broadening.

### Preparation of cassava pulp microcrystalline cellulose (CP-MCC)

CP-MCC was extracted from cassava bagasse as in a previous study with some modifications [31, 34]. Two hundred milliliters of 2% sodium hydroxide was added to 100 g of cassava bagasse. The solution was heated and stirred constantly at 85°C for 2 h. Next, it was rinsed with distilled water until the pH was neutral, and then 2000 ml of 6% sodium hypochlorite was added, heated and stirred constantly at 80°C for 4 h. The solution was rinsed with distilled water until the pH was neutral. Hydrochloric acid solution (2 mol/L) was added to the bleached cassava bagasse, heated and stirred constantly at 80°C for 2 h. The mixture was rinsed with distilled water until the pH was neutral. Finally, the obtained CP-MCC was dried in a hot-air oven at 65°C for 24 h, ground with a laboratory blender and stored in a desiccator.

### Preparation of CP-MCC butyrate as a filler

The CP-MCC structure was esterified as reported previously but with some modifications [20, 31]. First, a lithium chloride solution in dimethyl acetamide (DMAc/LiCl) was prepared at 8% w/v, and 50 mL was added to 2 g of CP-MCC powder. Then, the solution was heated and stirred constantly at 60°C until the powder was completely dissolved. Then, 1.35 g of methylaminopyridine (DMAP) catalyst and 13 mL of butyl chloride modifying agent were added, heated and stirred constantly at 90°C for 3 h. Next, the CP-MCC butyrate was precipitated by adding 7 volumes of 95% ethanol, filtered and rinsed with distilled water. Finally, the obtained CP-MCC butyrate was dried in a hot-air oven at 60°C for 24 h and stored in a desiccator.

### Characterization of CP-MCC butyrate

#### Determination of the percentage of weight increase

The percentage of weight increase (%*WI*) was calculated as

$$\%WI=\frac{W_{f}-W_{i}}{W_{i}}\times 100$$ (1)

where *W*_*i*_ is the weight of the dried initial cellulose sample (g) and *W*_*f*_ is the weight of the modified cellulose sample (g).

#### Dissolution test of CP-MCC butyrate in various solvents

The solubilities of CP-MCC and CP-MCC butyrate were tested in water and the five common solvents chloroform, toluene, tetrahydrofuran, acetone and ethanol by dissolving 25 mg of the sample in 3 ml of each solvent in screw-threaded, borosilicate-glass test tubes with polytetrafluoroethylene (PTFE)-faced rubber cap liners (Pyrex^®^, Tewksbury, MA, USA).

#### Studies of the CP-MCC and CP-MCC butyrate morphologies by scanning electron microscopy

The CP-MCC and CP-MCC butyrate were dried for 12 h in a hot air oven at 105°C and taped to a carbon stub. The samples were sputter-coated with a thin layer of gold to protect the surface from being charged by the electron beam under operating conditions using a sputtering power of 650 V, 200 mA, a glow discharge current of 200 mA, a sputtering rate of 12 nm/min and a distance of 35 mm in an argon atmosphere at 15 mA (Sputter Coater SCD 040, Balzers Union Ltd., Balzers, Principality of Liechtenstein). Scanning electron microscopy (SEM, model JSM-6610LV, JEOL Ltd., Tokyo, Japan) with an accelerating voltage of 15.0 kV was used to study the morphologies of CP-MCC and CP-MCC butyrate.

#### Fourier transform infrared spectroscopy (FT-IR)

The samples were mixed with KBr (1:100 by weight), and Fourier transform infrared (FTIR) spectroscopy was used to analyze the functional groups and chemical structures of the CP-MCC and CP-MCC butyrate (Nicolet NEXUS 670, Thermo Nicolet, Thermo Scientific Co., Madison, WI, USA); spectra were obtained over the range 4000–500 cm^-1^ with 64 scans and a resolution of 2 cm^-1^.

#### PHB_A-04_/CP-MCC butyrate biocomposite film preparation

The ratios of PHB_A-04_/CP-MCC butyrate were varied as 100:0, 95:5, 90:10, 85:15, 80:20 and 0:100 according to ASTM protocol D882-91. Biocomposite films of the PHB and CP-MCC butyrate were prepared by solvent casting. Chloroform was used as the casting solvent to dissolve both PHB and CP-MCC, and a Pyrex glass tray (Pyrex, Corning Incorporated, NY, USA) was used as the casting surface [35]. The solution was mixed with a vortex device and poured into a glass tray, and the chloroform was allowed to evaporate in the fume hood at room temperature. The obtained biocomposite films were left at room temperature for one week to crystallize. The polymer concentration in chloroform (1% w/v) and the volume of the polymer solution were used to control the thicknesses of the films. The thin films had thicknesses of 0.05 mm, as measured with a caliper (Model 500–175: CD-12C, Mitutoyo Corporation, Kawasaki-shi, Kanagawa, Japan). At least five film samples were cut (50 x 150 mm) for analysis and stored in desiccators at room temperature for one month to reach crystallization equilibrium.

### Characterization of PHB_A-04_/CP-MCC butyrate biocomposite films

#### Morphological analysis

The morphologies of the PHB_A-04_/CP-MCC butyrate films were observed with SEM (JSM-6610LV, JEOL Co. Ltd., Tokyo, Japan) as described above.

#### Thermal analyses by differential scanning calorimetry (DSC) and thermogravimetric analysis (TGA)

Thermal analyses using 10 mg of each sample were performed with a DSC apparatus (DSC 204 F1 Phoenix^®^, NETZSCH Thermal Analysis, NETZSCH-Gerätebau GmbH, Selb, Germany). Before thermal analysis, the thermal history of the sample was removed by heating it from ambient temperature to 180°C at 10°C/min and maintaining it at 180°C for 5 min before cooling to -50°C. The sample was then heated to 180°C at a rate of 10°C/min. The intersection between a tangent from the farthest point of an endothermic peak and the baseline was designated the melting peak temperature (T_m_). After heating the quenched sample, the glass transition temperature (T_g_) was calculated by extrapolating the midpoint of the heat capacity difference between the glassy and viscous states. Thermogravimetric analyses (TGA) were performed with a TGA 7 instrument (Perkin-Elmer Inc., Waltham, MA, USA). Each sample (10 mg) was heated from 30°C to 800°C at 10°C/min under a nitrogen atmosphere with a flow rate of 50 mL/min.

### Analysis of the mechanical properties of PHB_A-04_/CP-MCC butyrate biocomposite films

The mechanical properties of PHB_A-04_/CP-MCC butyrate biocomposite films were determined with a universal testing machine (H10KM, Wuhan Huatian Electric Power Automation Co., Ltd., Wuhan, China). The films (1 × 10 cm) were prepared according to the ASTM protocol D882-91. The crosshead was moved at a rate of 10 mm/min. The following mechanical data were determined: elongation at break point (%), stress at maximum load (MPa), and Young’s modulus (MPa). All of the data were reported as the result of three independent experiments with at least fifteen samples in total and expressed as the mean values ± standard deviations (SD).

#### Degradability in soil

The biodegradabilities of the biocomposite films were tested as described by Rizzarelli et al. and Phetwarotai et al. with some alterations [36, 37]. Nine samples with each biocomposite film ratio were used. A 1.5 cm × 1.5 cm biocomposite film placed in a mesh bag (8 cm × 4 cm) containing fertilized soil (inorganic nitrogen 0.35%, inorganic phosphorus 0.18%, inorganic potassium 1.37%, soil electrical conductivity 1.2 deciemens/m, soil organic matter 7.71%, pH 6.69) was buried in a plastic box containing fertilized soil with small holes perforated around the sides and bottom at depths of 20−30 cm from the soil surface; the system was held at room temperature (20−28°C), with 80–100% humidity and a pH of 6.69. Watering was performed every day for 24 days to keep the soil moisture level steady, and this was monitored with a digital moisture meter. The pH and temperature were monitored throughout the experiment. Samples of the buried film were collected every 7 days for 3 months, washed with distilled water, dried at 65°C for 24 h, and then stored in desiccators until the weight was stable. To investigate the degree of degradation, the collected samples were analyzed by considering the physical appearance, surface morphology and percentage of weight loss. The experiment was carried out independently and repeated 3 times.

#### Degradation of PHB_A-04_/CP-MCC butyrate biocomposite films studied by scanning electron microscopy

The surface morphologies of the PHB_A-04_/CP-MCC butyrate biocomposite films were observed before and after 56 days of degradation with a scanning electron microscope (SEM, JSM 6480, JEOL, Tokyo, Japan) operating at 15 kV. Before testing, each sample was rinsed with distilled water and dried in a vacuum at 40°C until it reached a consistent weight. To avoid surface charges generated by the electron beam, the surfaces of the samples were coated with gold prior to investigation.

#### Weight loss calculation

The weight loss of the PHB_A-04_/CP-MCC butyrate biocomposite film was determined by deducting the weight of the film sample after burial in the soil test from the beginning weight at regular intervals (7 days). In the soil burial test, the degradation rate was determined by the weight losses of the film samples. The following equation was used to calculate the weight loss percentage:

$$\text{Weight}\;\text{loss}(\%)=\frac{W_{i}-W_{f}}{W_{i}}\times 100$$ (2)

Where *W*_*i*_ is the initial weight of the sample before testing (g) and *W*_*f*_ is the final weight of the sample after testing (g). These data were calculated by applying the integrated first order kinetic equation [38, 39]

$$\text{ln}\frac{W_{f}}{W_{i}}=kt$$ (3)

where the time is expressed in days and *k*, the first order kinetic rate constant, is expressed in days^-1^. The half-life of the biocomposite, T12, was calculated with the following equation [38, 39]:

t12=ln(2)k (4)

#### Data analysis

All of the data presented in this paper were the results obtained from three independent experiments and were described as the mean values ± standard deviations (SDs). Analysis of variance (one-way ANOVA) followed by Duncan’s test for testing differences among means was conducted with SPSS version 22 (IBM Corp., Armonk, NY, USA). Differences were considered significant at P < 0.05.

## Results and discussion

### Cassava pulp compositions

The chemical composition of the CP was 15.63% (w/v) holocellulose, 11.05% (w/v) α-cellulose, 12.0% (w/v) lignin, 3–5% (w/v) ash and 60.10% (w/v) starch. According to Matsui et al. (2004), the chemical composition of CP varies between 40–60% starch, 15–50% cellulose, and other components, such as proteins and fat. Moreover, the compositions of the CP varied with changes in the species, cultivation location, cultivation conditions, harvest period and production process [28].

### Characterization of PHB produced by *C*. *necator* A-04

**Fig 1:**
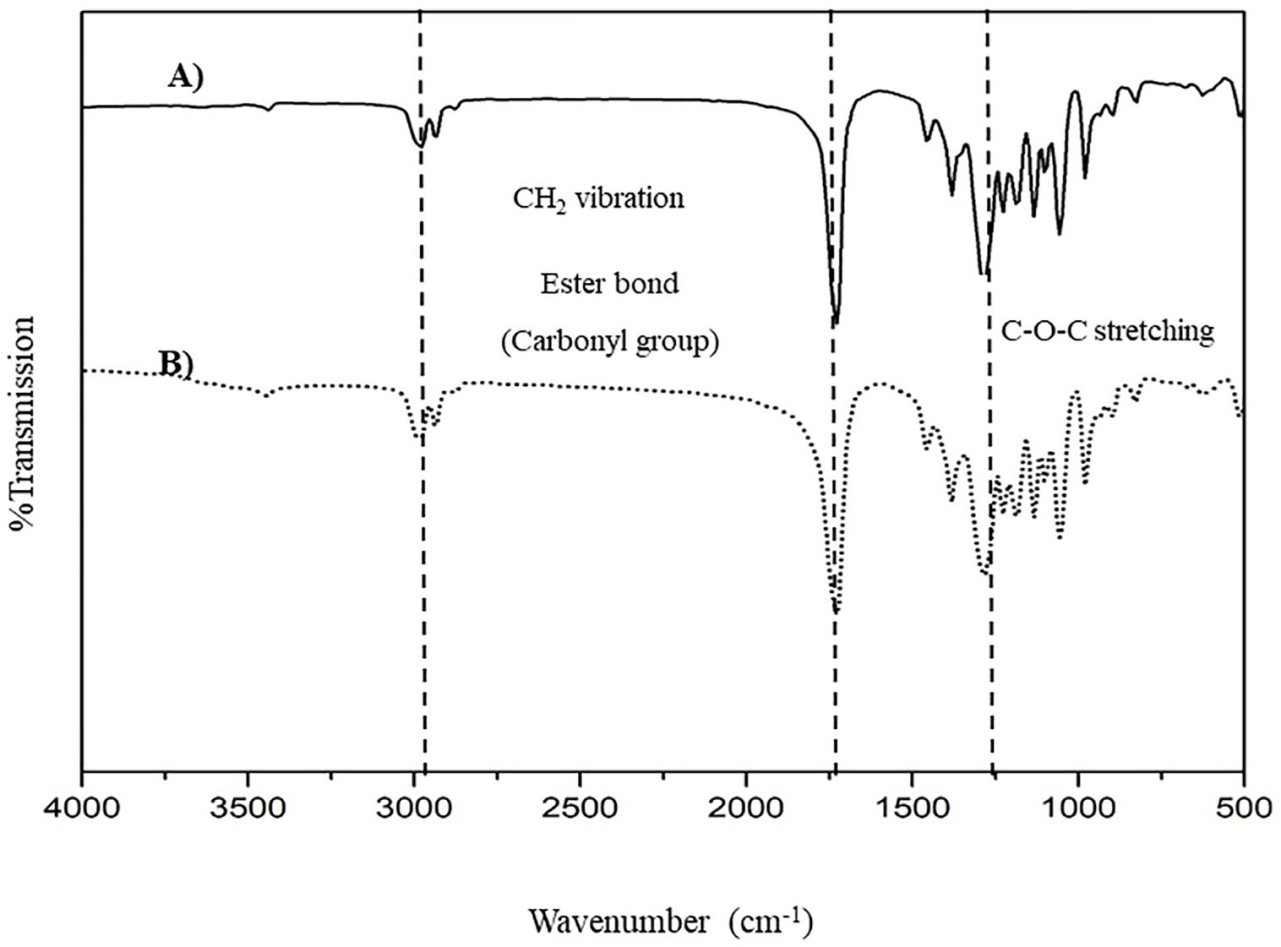
FTIR spectra of (A) PHB produced from *C*. *necator* strain A-04 and (B) commercial PHB (Sigma‒Aldrich Crop.).

After 60 h, the maximum total cell mass was 7.4 ± 0.4 g/L, and a maximum PHB content of 4.4 ±0.5 g/L was obtained, which represented a 59.7 ± 5.9 wt% yield. The extracted and purified PHB was white and fluffy. The functional groups of the PHB product and commercial PHB (Sigma‒Aldrich Corp.) were determined by FTIR, as shown in Fig 1. The spectrum of the extracted PHB exhibited peaks at 2981 cm^-1^, 1728 cm^-1^ and 1284 cm^-1^, which represented CH stretching vibrations, ester carbonyl bonds and C-O-C stretching, respectively [40, 41]. The ^1^H NMR spectrum was also examined to confirm that the polymer produced by *C*. *necator* A-04 was PHB (S1 Fig). The weight-average molecular weight (M_W_), number-average molecular weight (M_N_) and polydispersity index (PDI) of the PHB_A-04_ were 3.14×10^5^ Da, 1.06×10^5^ Da and 2.9, respectively (Table 1). The results of the DSC and TGA analyses are shown in Table 2. The T_g_, T_c_, T_m_, and T_d_ values of PHB_A-04_ were similar to those of commercial PHB. Therefore, it was confirmed that the extracted polymer was PHB.

**Table 1:** Mechanical data determined with a universal testing machine for commercialized PHB, produced PHB and produced PHB_A-04_/CP-MCC.

Samples	Mechanical properties		
	Young’s modulus	Tensile strength	Elongation at break
**filler**			
CP-MCC butyrate	924 ± 97.2	20.9 ± 3.2	2.7 ± 0.8
**major matrix**			
PHB_A-04_	1315 ± 54.3	14.7 ± 1.6	1.7 ± 0.3
**biocomposite**			
PHB_A-04_/CP-MCC butyrate			
95:5	1417 ± 241.8	24.3 ± 2.1	2.6 ± 0.1
90:10	1142 ± 272.0	18.2 ± 1.1	2.7 ± 0.5
85:15	1425 ± 193.8	16.0 ± 0.3	2.1 ± 0.2
80:20	1186 ± 358.9	15.0 ± 4.3	1.9 ± 0.7

**Table 2:** Thermal data determined by thermal gravimetric analysis (TGA) and differential scanning calorimetry (DSC) for commercialized PHB, produced PHB and produced PHB_A-04_/CP-MCC at a rate of 10°C/min.

Samples	Thermal properties						
	T_M_ (°C)	T_C_ (°C)	T_G_ (°C)	T_D_ at 50% (°C)	△H_C_ (j/g)	△H_M_ (j/g)	X_C_ (%)
**filler**							
Unmodified CP-MCC	ND	ND	74.0	336.1	ND	ND	ND
CP-MCC butyrate	ND	ND	115.6	352.1	ND	ND	ND
**major matrix**							
PHB (Sigma‒Aldrich)	175.4	48.0	3.5	263.6		99.0	68.0
PHB_A-04_	172.8	55.7	3.6	261.0		99.7	68.3
**biocomposite**							
PHB _A-04_/CP-MCC butyrate							
95:5	176.0	75.3	2.8	263.3		88.0	63.4
90:10	173.2	74.2	2.8	260.2		81.7	62.2
85:15	168.0	74.4	0.5	261.8		82.2	66.3
80:20	157.8	81.9	-3.4	262.6		73.6	63.0

ND = not detected

### FTIR spectrum, solubility, morphology and thermal properties of CP-MCC and CP-MCC butyrate

#### Functional groups determined by FTIR

**Fig 2:**
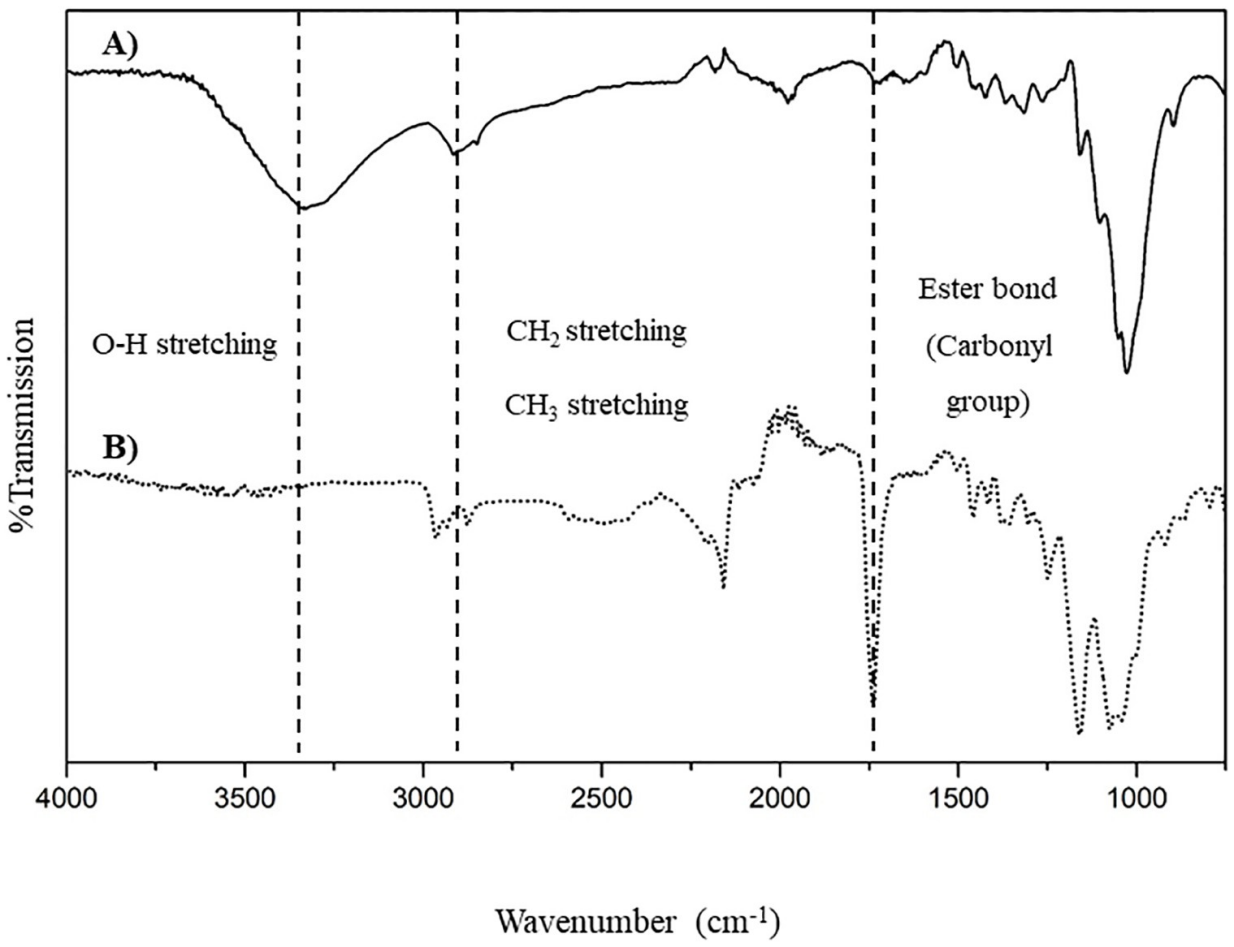
FTIR spectra of (A) native CP-MCC and (B) CP-MCC butyrate.

Functional group analyses of the CP-MCC and CP-MCC butyrate are shown in Fig 2. The FTIR spectra showed distinct infrared absorptions for CP-MCC and CP-MCC butyrate: 1) peaks at 3300–3500 cm^-1^ indicated O-H stretching vibrations, which were found in CP-MCC structures, and the percent transmittance values increased when the CP-MCC was structurally modified and the number of hydroxyl groups was decreased; 2) peaks at 1739 cm^-1^ indicated C = O stretching vibrations of esters, which were found only in CP-MCC butyrate because the hydroxyl groups were esterified with carbonyl groups supplied by the modifying agent. Therefore, these peaks were not detected in the CP-MCC structure because there were no esterified hydroxyl groups. 3) The peaks in the range 2875–2916 cm^-1^ were caused by CH_3_ and CH_2_ stretching vibrations, which were found in both the CP-MCC and CP-MCC butyrate structures. However, the percent transmittance was increased for the CP-MCC butyrate structure due to the replacement of hydroxyl groups by aliphatic chains from the modifying agent [20, 31]. This result was consistent with those of a previous study that reported esterification of cellulose from waste cotton fabric and pineapple leaves. Based on the spectral differences for CP-MCC and CP-MCC butyrate, it was concluded that the CP-MCC structure was modified successfully.

#### Solubility properties

The solubilities of CP-MCC butyrate and CP-MCC were compared in the common organic solvents acetone, chloroform, ethanol, tetrahydrofuran and toluene. CP-MCC was insoluble in all five solvents since the cellulose structure contained many hydroxyl groups. As a result, the cellulose had interchain and intrachain hydrogen bonds and was highly crystalline. Therefore, it could not be dissolved in the solvents, whereas the structure of CP-MCC butyrate had been modified by substitution with aliphatic chains, resulting in hydrophobicity and decreased hydrogen bonding. Thus, CP-MCC butyrate was soluble in acetone, chloroform and tetrahydrofuran, but the solubility in toluene depended on the degree of displacement with aliphatic chains. Ratanakamnuan et al. (2012) tested the solubilities of MCC modified with butyl chloride, caproyl chloride and lauroyl chloride. MCC butyrate was soluble in both polar (acetone) and nonpolar (toluene, chloroform, N,N-dimethyl acetamide and tetrahydrofuran) solvents, while MCC caproate and MCC laurate were moderately to highly soluble in nonpolar solvents. The high solubility of MCC butyrate resulted from the high degree of aliphatic chain substitution. In contrast, the solubilities of MCC caproate and MCC laurate decreased with higher levels of aliphatic chain incorporation, because the long aliphatic chains caused crystallization, which resulted in decreased solubility [20].

#### Morphology

**Fig 3:**
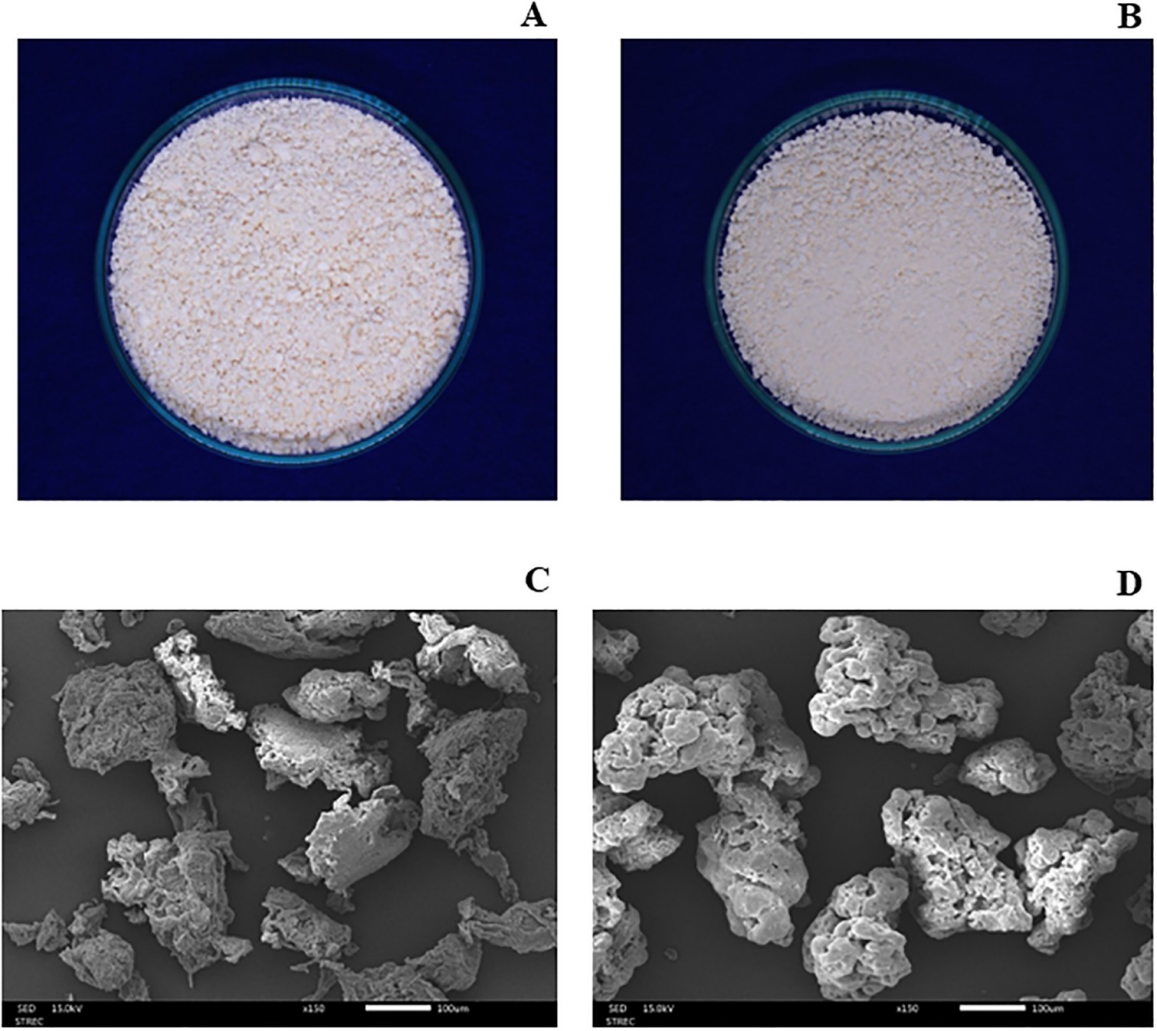
Physical appearance of **(A)** native CP-MCC powder and **(B)** CP-MCC butyrate powder. Morphologies determined by SEM for **(C)** native CP-MCC and **(D)** CP-MCC butyrate.

CP-MCC appeared as a fine and creamy powder, whereas the esterified CP-MCC was a fine and slightly white powder, as shown in Fig 3A and 3B, respectively. The morphology of CP-MCC was characterized by SEM, and the particle sizes were estimated with ImageJ software (S2 Fig). CP-MCC presented flat and long filaments arranged in bundles with smooth surfaces. The average particle size was approximately 160.9 ± 56.4 µm. The particle sizes of prepared CP-MCC depended on the cellulose extraction conditions, such as the temperature, method, type and concentration of acid, ratio between acid and cellulose and type of raw material, all of which affected the crystal sizes, crystallinity, thermal stability and mechanical properties of the microcrystalline cellulose [42], whereas the CP-MCC butyrate particles were larger than those of CP-MCC. The surfaces of the CP-MCC butyrate particles were rough and included many round particles with attached butyl aliphatic chains that had replaced the hydroxyl groups of cellulose. The particle sizes were approximately 206.6 ± 69.1 µm. The CP-MCC and CP-MCC butyrate morphologies determined by SEM are shown in Fig 3C and 3D, respectively.

#### Thermal properties

**Fig 4:**
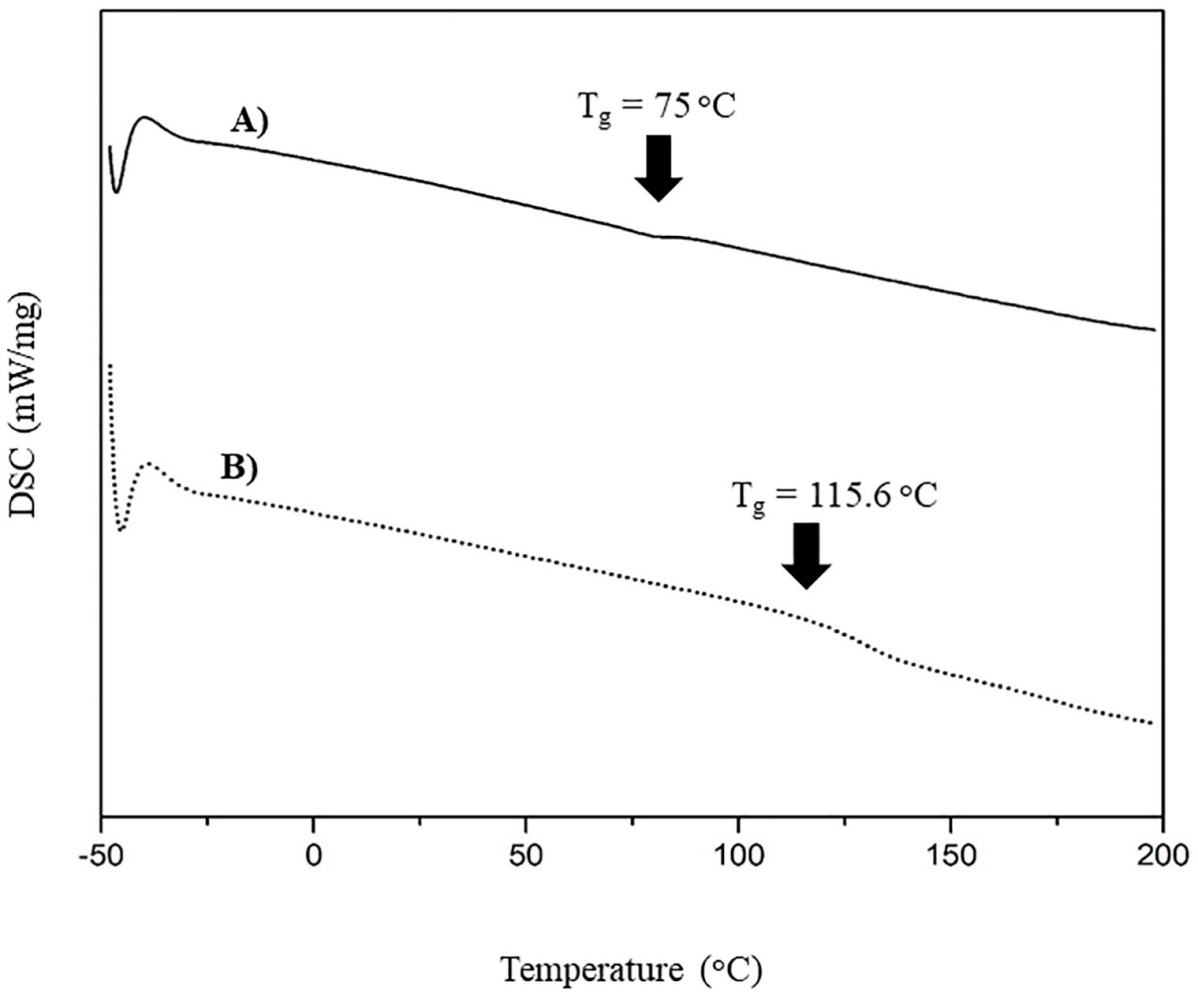
DSC thermograms of (A) native CP-MCC and (B) CP-MCC butyrate.

**Fig 5:**
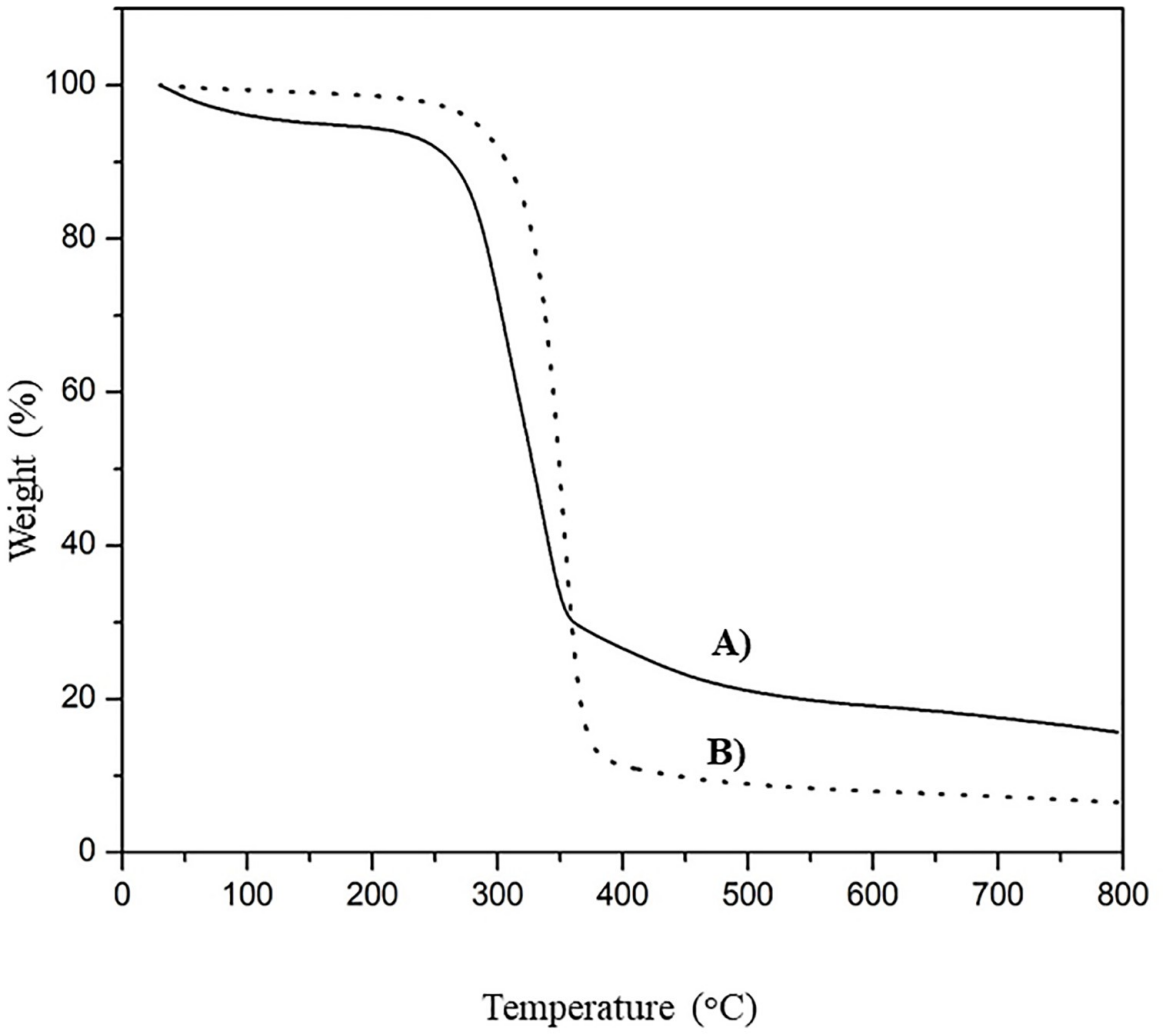
TGA thermograms of (A) native CP-MCC and (B) CP-MCC butyrate.

The DSC thermograms illustrating the glass transition temperatures (T_g_) and melting temperatures (T_m_) and the TGA thermograms illustrating the degradation temperatures (T_d_) of CP-MCC and CP-MCC butyrate are shown in Figs 4 and 5, respectively. The results showed that CP-MCC and CP-MCC butyrate did not present T_m_ because cellulose had a T_m_ that was close to the T_d_. This suggested that the cellulose modification did not cause a change in T_m_ in the range below 200°C. The T_g_ of CP-MCC was 74.0°C, whereas the T_g_ of CP-MCC butyrate was 115.6°C, which was higher because of the aliphatic chains substituted into the cellulose structure. As a result, the CP-MCC butyrate particles became larger. This was consistent with the morphology results determined by SEM, which showed that the modified cellulose had larger particles. More thermal energy was needed to cause movement of the CP-MCC butyrate molecules. Therefore, the T_g_ of CP-MCC butyrate was increased.

The TGA thermogram of CP-MCC showed that there were three weight loss phases at different temperatures: 1) at 50−100°C, there was a weight loss of approximately 10% caused by the loss of water or moisture from CP-MCC; 2) at 250−350°C, there was a weight loss of approximately 72.33% due to the degradation of cellulose; and 3) at temperatures above 400°C, there were weight losses of approximately 15% due to degradation of other impurities, such as lignin and ash. In contrast, the TGA thermogram of CP-MCC butyrate showed that there were two weight loss phases at different temperatures: 1) at 150°C, there was a weight loss of approximately 5% due to decomposition of the aliphatic chains derived from the agent used to modify CP-MCC butyrate; 2) at 250−370°C, there was a weight loss of 87.83% due to the decomposition of cellulose. In comparing the weight losses for CP-MCC and CP-MCC butyrate, the weight loss at 50−100°C occurred only with CP-MCC. This arose because the CP-MCC structure contained many hydroxyl groups generating hydrogen bonds with water; thus, it was hydrophilic. After modifying the cellulose, the presence of the aliphatic chains and carbonyl groups in the ester substituents resulted in hydrophobicity. Therefore, weight loss at 50−100°C was not observed for the CP-MCC butyrate structure. It was concluded that the structure of CP-MCC was changed after modification. In comparing the T_d_ values of CP-MCC and CP-MCC butyrate, the T_d_ of CP-MCC was 336.05°C. After modification of the cellulose structure, the T_d_ was increased to 352.13°C [43]. Furthermore, other studies investigated the effects of different aliphatic chain lengths in the fatty acids used to modify the MCC structure and determined whether T_d_ was increased due to rearrangements of the aliphatic chains that resulted in higher crystallinity [44–46].

### Effect of the CP-MCC butyrate content on the mechanical properties of PHB_A-04_/ esterified CP-MCC butyrate biocomposite films

Mechanical properties such as the Young’s modulus, tensile strength and elongation at break were investigated for PHB_A-04_/CP-MCC butyrate biocomposite films made with different ratios, as shown in Table 1. The proportions of CP-MCC butyrate in the biocomposite films were varied at 0, 5, 10, 15, 20 and 100%. For the PHB film, the Young’s modulus was 1315 ± 54.3 MPa, the tensile strength was 14.7 ± 1.6 MPa and the elongation at break was 1.7 ± 0.3%, whereas the Young’s modulus, tensile strength and elongation at break for the CP-MCC butyrate film were 924 ± 97.2 MPa, 20.9 ± 3.2 MPa, and 2.7 ± 0.8%, respectively. The Young’s moduli were similar for all proportions in the biocomposite films. For biocomposite films containing 5% and 10% CP-MCC butyrate, there was a significant increase in tensile strength compared with that of the CP-MCC butyrate film. The tensile strength was increased because CP-MCC butyrate formed a hard phase dispersed in the PHB matrix, which resulted in the absorption of external stress. Therefore, the tensile strength was increased. This result was supported by the work of Morelli et al. (2016), who examined the mechanical properties of polybutylene adipate-co-terephthalate (PBAT) and modified nanocrystalline cellulose composite films. It was revealed that there was a 40% increase in tensile strength [47]. The elongation at break of the biocomposite films containing 5% and 10% CP-MCC butyrate were increased substantially compared with those of PHB films and decreased when more than 10% CP-MCC butyrate was added, since the addition of excess CP-MCC butyrate resulted in loose aggregation of attached CP-MCC butyrate polymers. These generated points of stress concentration in the biocomposite films and affected the distribution of external stress. As a result, the elongation at break was decreased. This result was similar to those of Pinheiro et al. (2017), who compared the mechanical properties of nanomaterials for polybutylene adipate-co-terephthalate (PBAT) and modified cellulose nanocrystals. They found that the elongation at break increased when modified nanocrystalline cellulose was added. However, the elongation at break decreased when the amount of modified nanocrystalline cellulose added was increased by 3−7% [48]. The factors affecting the mechanical properties of the biocomposites, in addition to the cellulose content, were the type and size of the cellulose, the preparation process, the method used for modifying the structure of the cellulose, the type of matrix and the dispersion and arrangement of cellulose in the polymer matrix [49].

### Fracture surfaces of PHB_A-04_/CP-MCC butyrate biocomposite films

**Fig 6:**
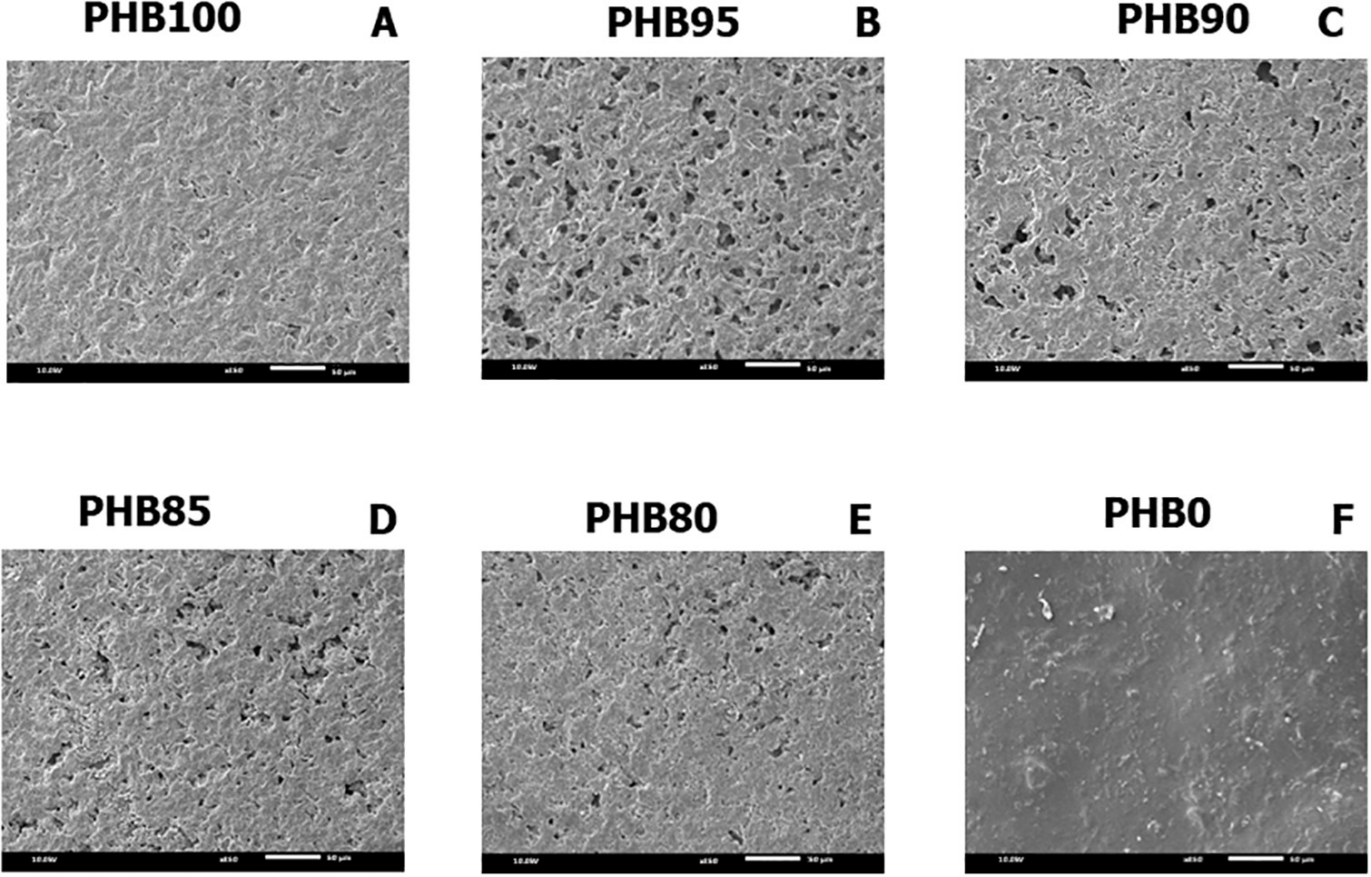
Film surface morphologies observed by SEM; (A) PHB_A-04_, PHB_A-04_/CP-MCC butyrate at (B) 95:5 (C) 90:10 (D) 85:15 (E) 80:20 and (F) CP-MCC butyrate films.

The PHB_A-04_/CP-MCC butyrate biocomposite films were opaque, hard and brittle. When the content of CP-MCC butyrate was increased, the biocomposite films became slightly yellow and brittle, whereas the CP-MCC butyrate film was more translucent and more brittle than the PHB film. The surface morphologies of the PHB_A-04_/CP-MCC butyrate biocomposite films were observed by SEM (Fig 6A–6F). The PHB film showed a rough surface with slight porosity, whereas the CP-MCC film had a rough surface. Small and short fibers were dispersed in the PHB matrix. When the content of CP-MCC butyrate was increased, the surface porosity of the biocomposite film decreased. There were also more small and short fibers dispersed on the surface. This indicated that the biocomposite material comprising PHB and CP-MCC butyrate was not formed homogeneously during film preparation.

### Thermal properties of the PHB_A-04_/CP-MCC butyrate biocomposite films

**Fig 7:**
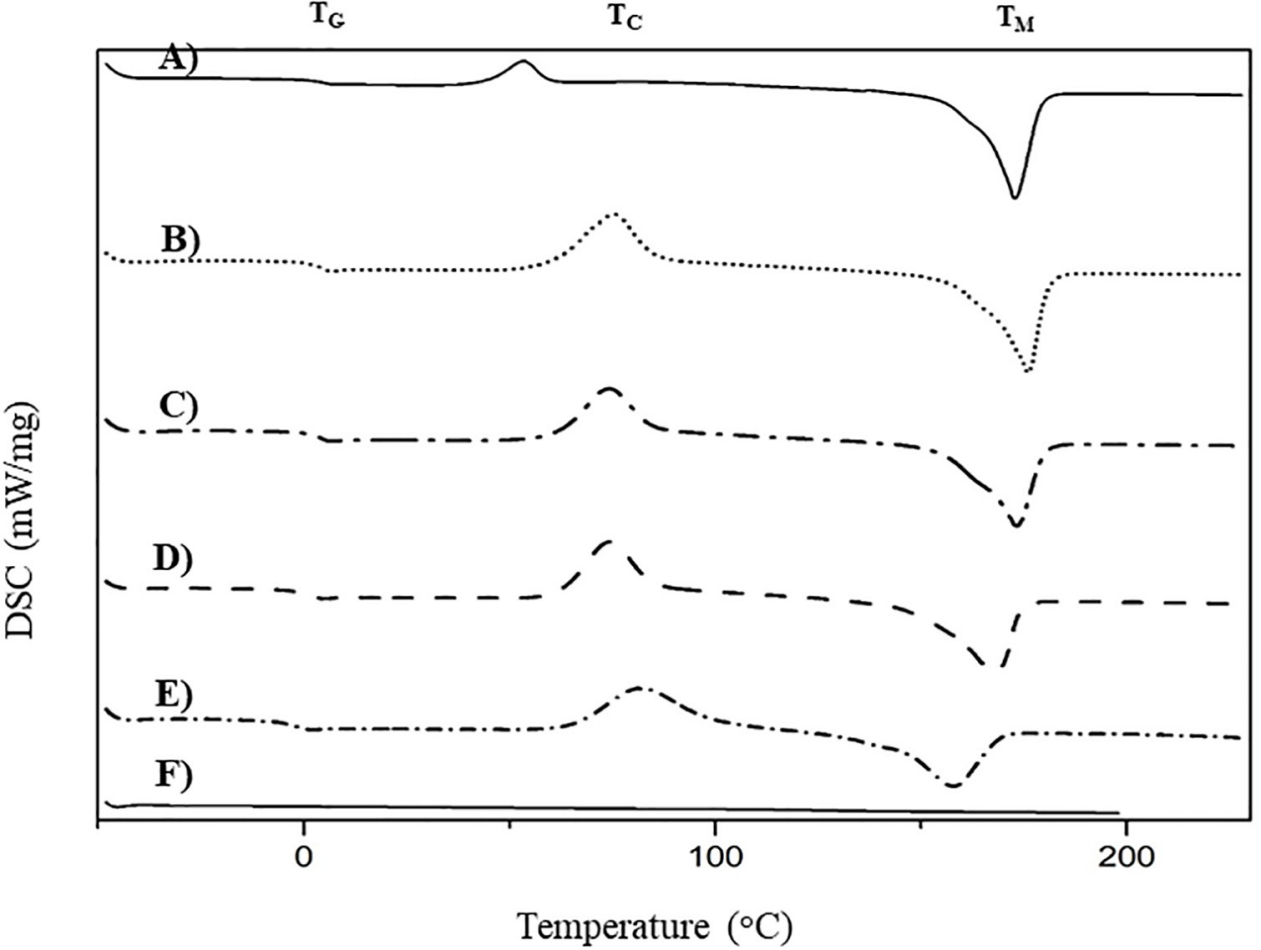
DSC thermograms of films; (A) PHB_A-04_, PHB_A-04_/CP-MCC butyrate at (B) 95:5 (C) 90:10 (D) 85:15 (E) 80:20 and (F) CP-MCC butyrate films.

**Fig 8:**
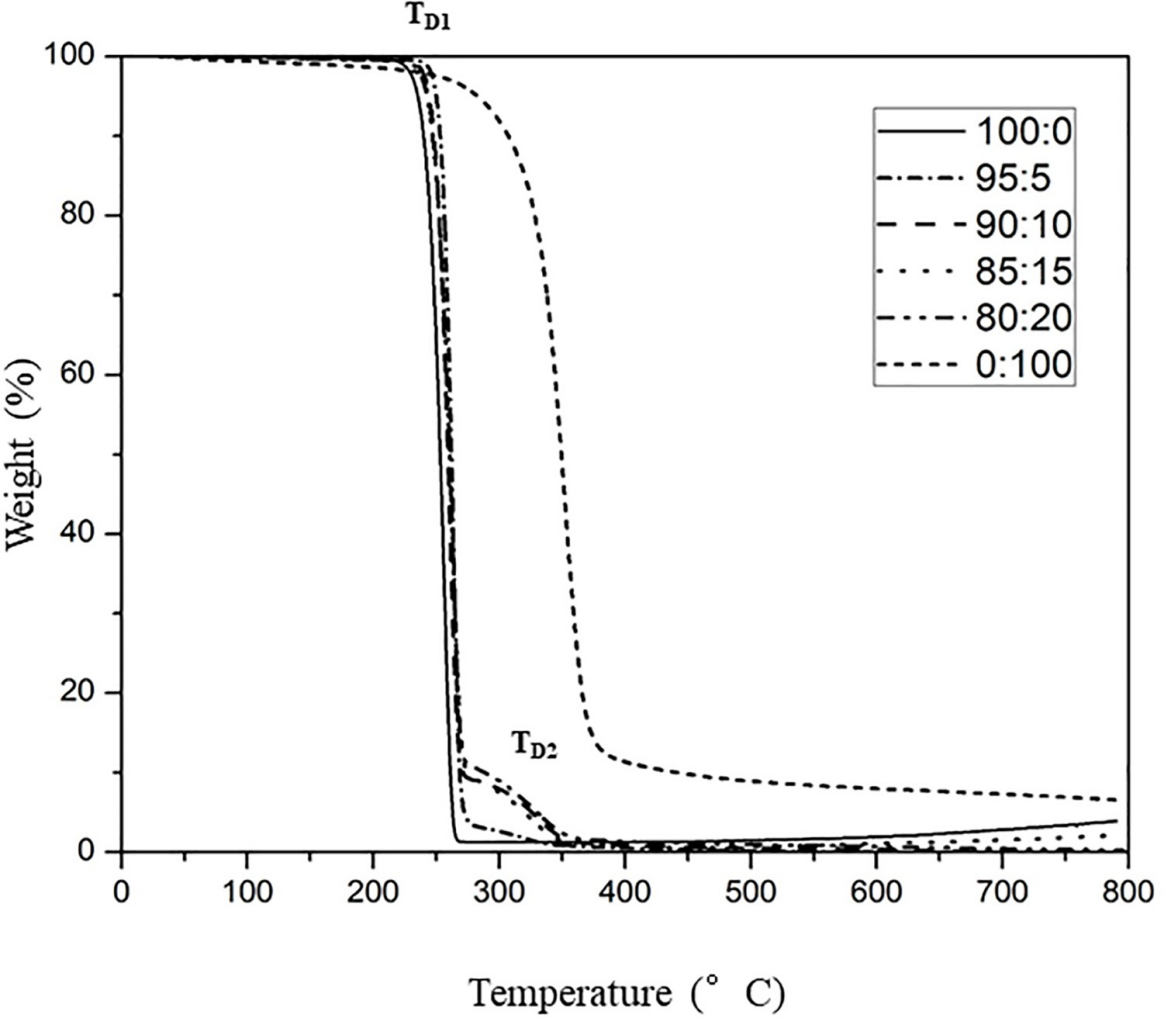
TGA thermograms of PHB_A-04_, PHB_A-04_/CP-MCC butyrate at 95:5, 90:10, 85:15, 80:20, and CP-MCC butyrate films.

The DSC thermograms illustrating the glass transition temperatures (T_g_) and melting temperatures (T_m_) of the PHB_A-04_ film and PHB_A-04_/CP-MCC butyrate films with 95:5, 90:10, 85:15, 80:20 ratios and the CP-MCC butyrate film are shown in Fig 7. The TGA thermograms illustrating the degradation temperatures (T_d_) at 50% weight loss for the PHB_A-04_ film, PHB_A-04_/CP-MCC butyrate films at 95:5, 90:10, 85:15, 80:20 and CP-MCC butyrate film are shown in Fig 8. Due to the thermal stability of CP-MCC butyrate, 100% (w/w) CP-MCC butyrate resulted in a higher T_d_ than PHB_A-04_ at 352.13°C. The T_d_ of PHB_A-04_ was approximately 261.0°C, and it showed a 98.54% weight loss at 275°C. As shown in Table 2, the ester groups grafted onto the cellulose structure increased the inter- and intramolecular hydrogen bonding and crystallinity of the cellulose, shifting the T_d_ of the PHB_A-04_/CP-MCC butyrate composites toward a higher temperature than that seen without the cellulose ester.

Table 2 provides a summary of the TGA and DSC data for the PHB_A-04_/CP-MCC butyrate composites, and the results indicated that the added cellulose fiber had no effect on the thermal degradation of PHB and P(HB-co-HV) [31, 50, 51]. As shown in Fig 8, all PHB_A-04_/CP-MCC butyrate composites had two T_d_ values: the major and first one was attributed to the slightly decreased T_d_ of PHB_A-04_ (weight loss of more than 80%), and the other was attributed to the T_d_ of CP-MCC butyrate at approximately 352.13 °C with a weight loss of 2–9% depending on the CP-MCC butyrate content. It was discovered that the T_m_ and T_g_ values of the composite films did not change significantly in the DSC thermograms of the PHB_A-04_/CP-MCC butyrate composites.

Increases in T_c_ were noted in all PHB_A-04_/CP-MCC butyrate composites, as shown in Table 2. All PHB_A-04_/CP-MCC butyrate composites showed endothermic transitions, which can only be attributed to the PHB_A-04_ phase because CP-MCC butyrate, like all other cellulose fibers, exhibits temperatures for gelatinization and breakdown rather than melting [52]. Moreover, the added CP-MCC butyrate had no effect on the T_g_ values.

### Effect of the CP-MCC butyrate content used as a filler in PHB_A-04_/CP-MCC butyrate biocomposite films on biodegradability

**Fig 9:**
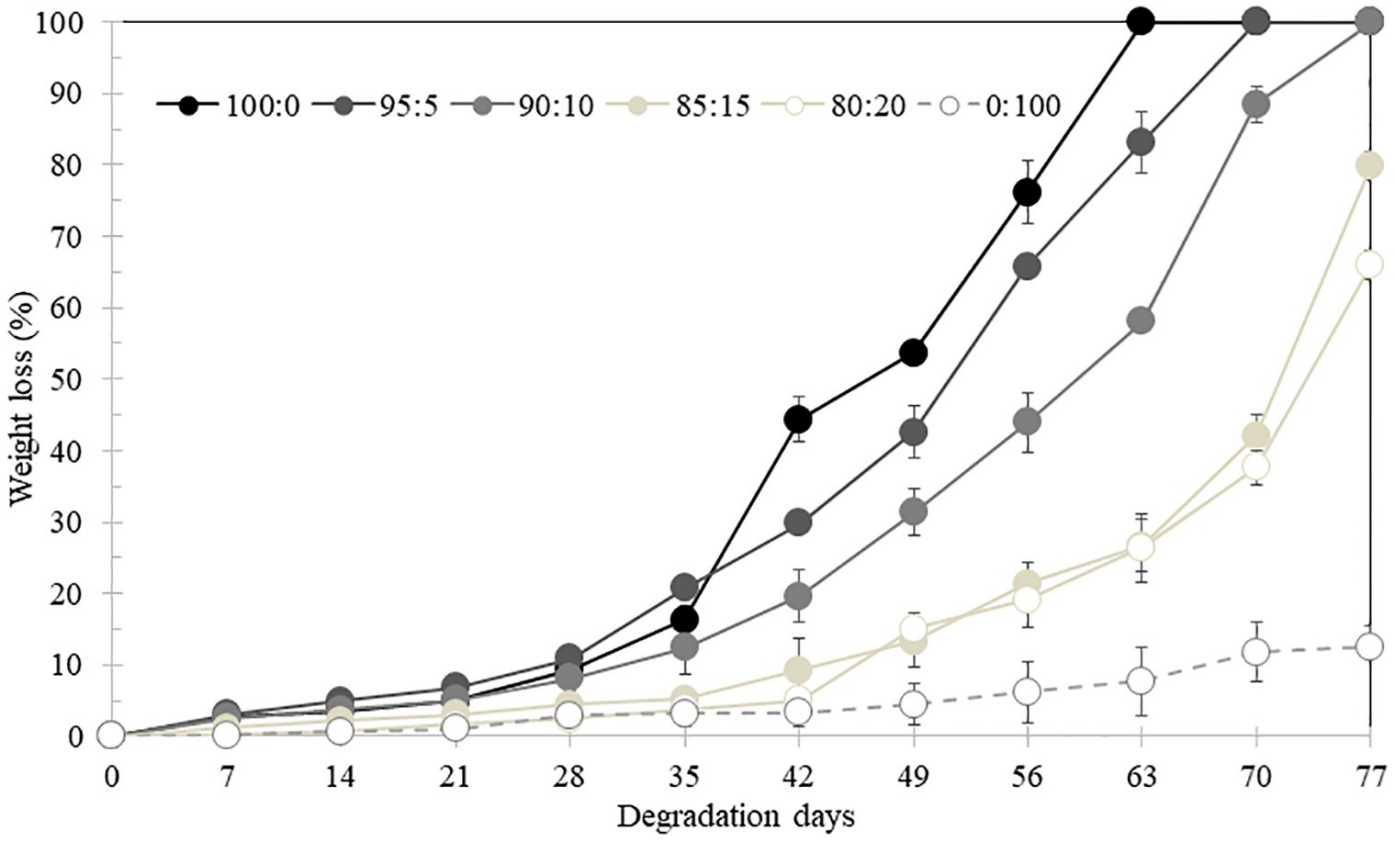
Percentage of weight lost by PHB_A-04_, PHB_A-04_/CP-MCC butyrate at 95:5, 90:10, 85:15, and 80:20 and CP-MCC butyrate films degraded in soil at the laboratory scale for 77 days.

Degradation of the PHB_A-04_/CP-MCC butyrate biocomposite films was investigated by burying them in fertilized soil with the pH kept constant at 5–7; the humidity of soil was kept at 80–100%, and the temperatures ranged between 20–28°C. Samples were collected every 7 days to determine the weight losses. The surface morphologies of the degraded films were characterized by SEM. The biodegradation rate was determined by calculating the weight loss of the degraded film. A linear regression plot of the film weight losses recorded every seven days for seventy-seven days provided a rate constant for degradation of the PHB_A-04_/CP-MCC butyrate biocomposite film. The percentage of weight lost by the biocomposite film is shown in Fig 9. These results revealed that weight loss was directly proportional to the days of degradation. The degradation data revealed very fast degradation with the soil burial method used. The linear section of the curve for the biocomposite was considered to indicate full degradation was accomplished. The degradation parameters determined were the rate constants *k*, half-life for degradation *T*_*1/2*_ and erosion rates, and these values are displayed in Table 3. The highest degradation rate was 1.2% per day, which corresponded to a *T*_*1/2*_ of 41 days found for the PHB film, whereas the lowest degradation rate was 0.1% per day, which corresponded to a *T*_*1/2*_ of 385 days found for the CP-MCC butyrate film. When the content of CP-MCC butyrate in the biocomposite film was increased, *k* varied inversely; among the various biocomposite film contents, 5% CP-MCC butyrate showed the highest degradation rate of 1.1% per day. The addition of CP-MCC butyrate to the biocomposite films decreased their biodegradabilities because hydroxyl groups were replaced by aliphatic chains and ester carbonyl groups were introduced by butyryl chloride and served as modifying agents, which increased the hydrophobicity of the modified CP-MCC. Therefore, this led to reduced water and moisture absorption by the biocomposite films. However, facile degradation in soils containing many microorganisms requires moisture. The degradation of the biocomposite films depended on the duration of biodegradability testing. When the duration was too short, degradation was not observed. Furthermore, the degradation conditions in the soil, such as the temperature, moisture, pH, ratio of carbon to nitrogen, and aeration; the thickness, molecular weight and morphology of the film; the more facile degradation of amorphous segments than crystalline segments; and the types of microorganisms present all affected degradation.

**Table 3:** Comparison of the degradation rates for PHB_A-04_/CP-MCC butyrate biocomposite films with CP-MCC butyrate contents of 0, 5, 10, 15, 20 and 100% (w/w).

Samples	Degradation parameters		
	k (% per day)	T1/2 (day)	Erosion rate [mg (cm^2^ day)^-1^]
**filler**			
CP-MCC butyrate	0.13	385	0.02
**major matrix**			
PHB_A-04_	1.24	41	0.51
**biocomposite**			
PHB_A-04_/CP-MCC butyrate			
95:5	1.14	44	0.38
90:10	0.94	54	0.33
85:15	0.54	93	0.19
80:20	0.47	107	0.12

**Fig 10:**
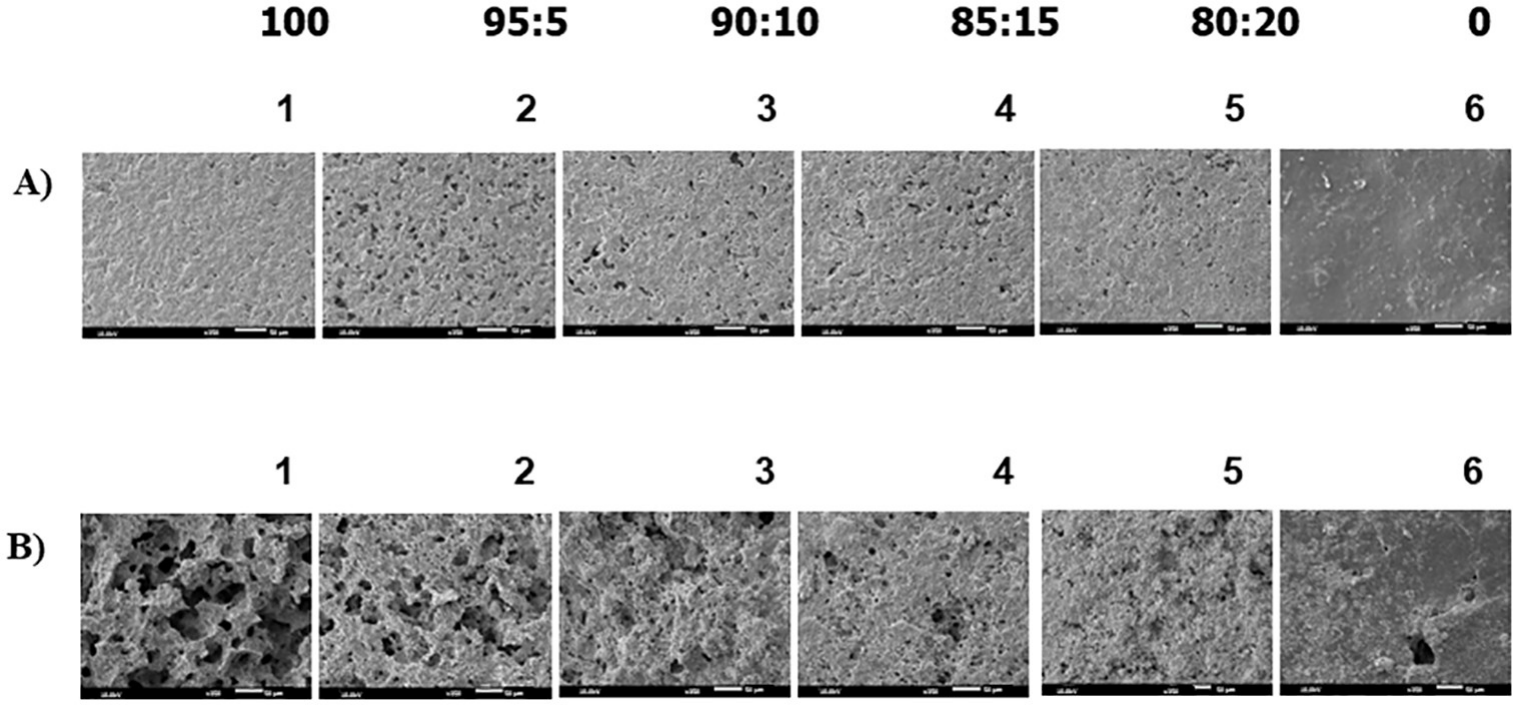
Film surface morphologies were observed by SEM for PHB_A-04_, PHB_A-04_/CP-MCC butyrate at 95:5, 90:10, 85:15, and 80:20 and CP-MCC butyrate films (A) before degradation and (B) after degradation for 56 days.

The physical appearances of biocomposite films degraded in soil are shown in S3 Fig. On day 35, the PHB film was darker and exhibited erosion at the edges and tiny pores on the surface, and the CP-MCC butyrate film had a slight break at the edge. For the biocomposite films with 5% and 10% CP-MCC contents, erosion occurred at the edges of the films, and pores were formed on the surfaces of the films. For the biocomposite films with 15% and 20% CP-MCC contents, changes in physical appearance due to erosion and fracture were observed on day 42. The morphologies of the degraded biocomposite films compared with those of the PHB film and CP-MCC film are shown in Fig 10. After degradation, the PHB film and the biocomposite films with 5% and 10% CP-MCC contents showed remarkable increases in the numbers of pores, whereas 15% and 20% contents of CP-MCC and CP-MCC butyrate in biocomposite films resulted in slightly more pores after degradation. This corresponded to the percentage of weight lost from the film during degradation. In the work of Jain and Tiwari (2015), *C*. *necator* was cultivated to produce PHB. Then, it was blended with cellulose acetate butyrate to prepare a film and tested for biodegradability in soil. The PHB film and the blended film with a 50:50 ratio showed 64.3% and 31.5% biodegradabilities, respectively. The surface of the blended film was hydrophobic. The percentage of biodegradability of the blended film was lower than that of the PHB film [52]. Based on all of the above, possible applications of the PHB_A-04_/CP-MCC butyrate biocomposites include use as disposable materials in medical device packaging, medical films, bread packaging, coffee cup sleeves, etc.

## Conclusion

Researchers are interested in PHB because of its desirable properties, including thermoplasticity, renewability, biodegradability, and biocompatibility. However, PHB is hard and brittle, which limits its application. To improve these physical properties, this research was designed to determine the physical properties and biodegradability of biocomposite films prepared from a PHB_A-04_ matrix and CP-MCC butyrate obtained from cassava bagasse, which is a renewable material serving as a natural filler (S4 Fig). The resulting biocomposite film showed that the PHB and CP-MCC butyrate did not blend homogenously, as observed by SEM. The T_D_ data presented two weight loss phases for PHB and CP-MCC butyrate, and the percentage of CP-MCC butyrate weight loss increased when the content of CP-MCC was increased. The T_d_ values of the biocomposite films increased, indicating increased thermal stability. For mechanical properties, the tensile strength and elongation at break increased for biocomposite films with CP-MCC butyrate contents of 5% and 10% because the CP-MCC butyrate was dispersed throughout the matrix phase. In the biodegradability tests in soil, a 5% CP-MCC butyrate content led to the highest biodegradability rate. The physical appearance showed fractured films, which were confirmed by the morphologies of the degraded films seen with SEM; these displayed increasing numbers of pores as degradation progressed. This confirmed that the biocomposite films maintained biodegradability.

## Supporting information

S1 Fig 500-MHz ^1^H NMR spectrum of PHB_A-04_ produced by *C*. *necator* A-04 in this study. (TIF)

S2 Fig Particle diameters for (A) native CP-MCC and (B) CP-MCC butyrate determined with SEM and ImageJ analysis. (TIF)

S3 Fig Time course photographs showing disintegration of PHB_A-04_/CP-MCC butyrate biocomposite films during laboratory-scale composting in a soil burial test conducted for 77 days. (TIF)

S4 Fig Schematic diagram for the preparation of esterified CP-MCC bytyrate to depict the method for preparing fibres to be compatible with solvent casting PHB_A-04_. (TIF)

## Abbreviations

ΔH_C_: enthalpy of crystallization
ΔH_M_: enthalpy of fusion
CDM: cell dry mass
CP: cassava pulp fibers
CP-MCC: cassava pulp fiber-microcrystalline cellulose
DSC: differential scanning calorimetry
GPC: gel permeation chromatography
M_N_: number-average molecular weight
M_W_: weight-average molecular weight
PHB: poly(3-hydroxybutyrate)
T_c_: crystallization temperature
T_d_: degradation temperature
T_g_: glass transition temperature
TGA: thermal gravimetric analysis
T_m_: melting temperature
X_C_ (%): percent crystallinity
