# Supplementary figures and images for: Valorization of agro-industrial waste from the cassava industry as esterified cellulose butyrate for polyhydroxybutyrate-based biocomposites
Source: PLoS One. 2023 Nov 22;18(11):e0292051. doi: 10.1371/journal.pone.0292051 (PMC10664873; doi:10.1371/journal.pone.0292051)

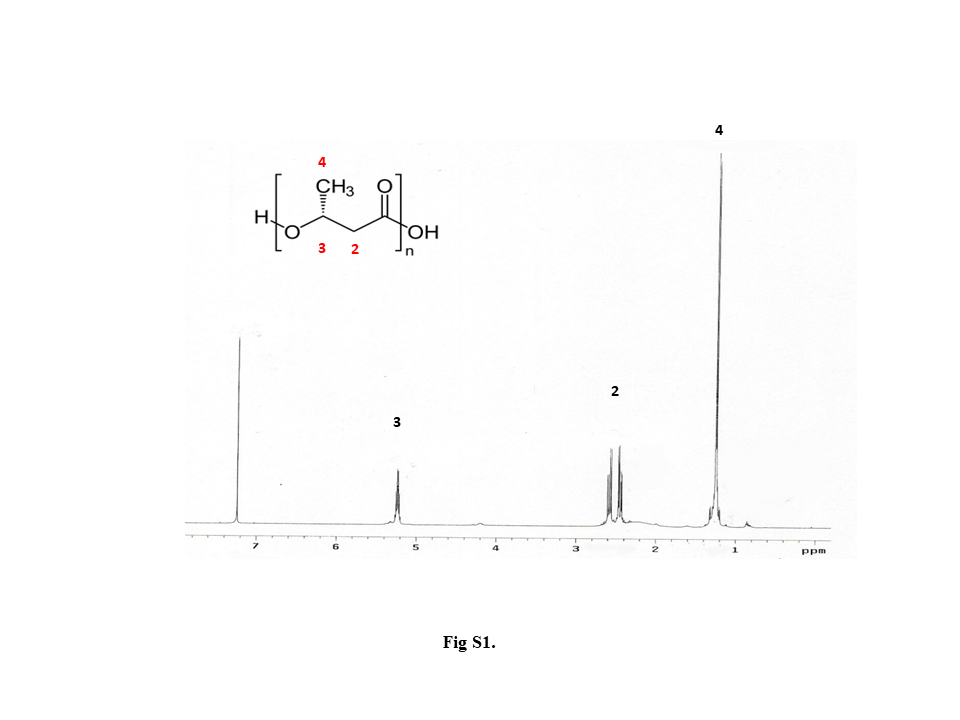

Supplement: S1 Fig — (TIF) [file pone.0292051.s001.TIF]

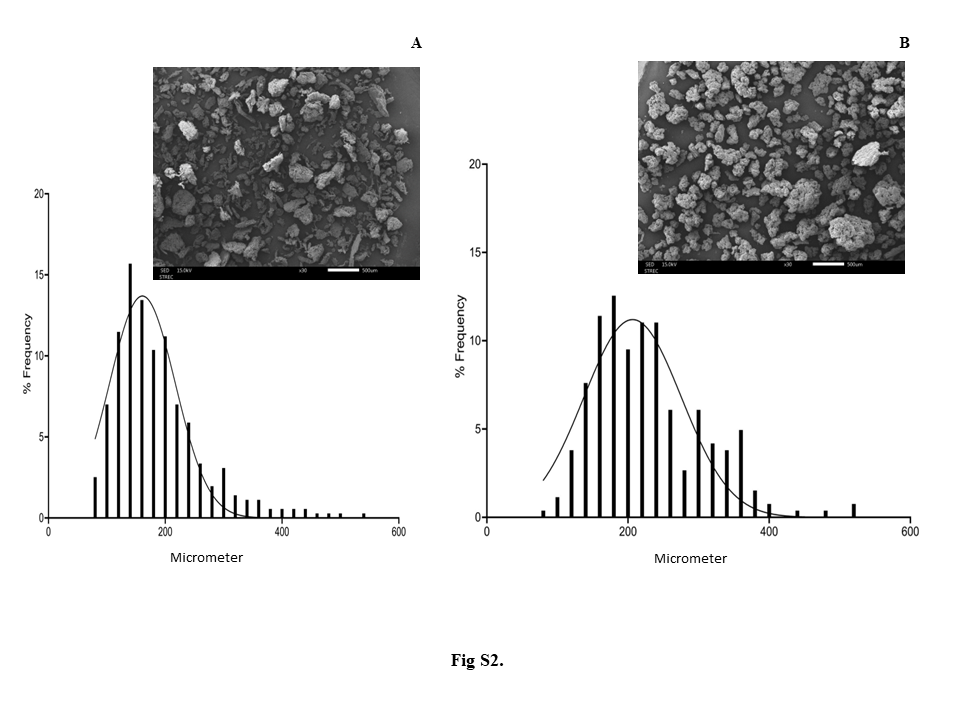

Supplement: S2 Fig — (TIF) [file pone.0292051.s002.TIF]

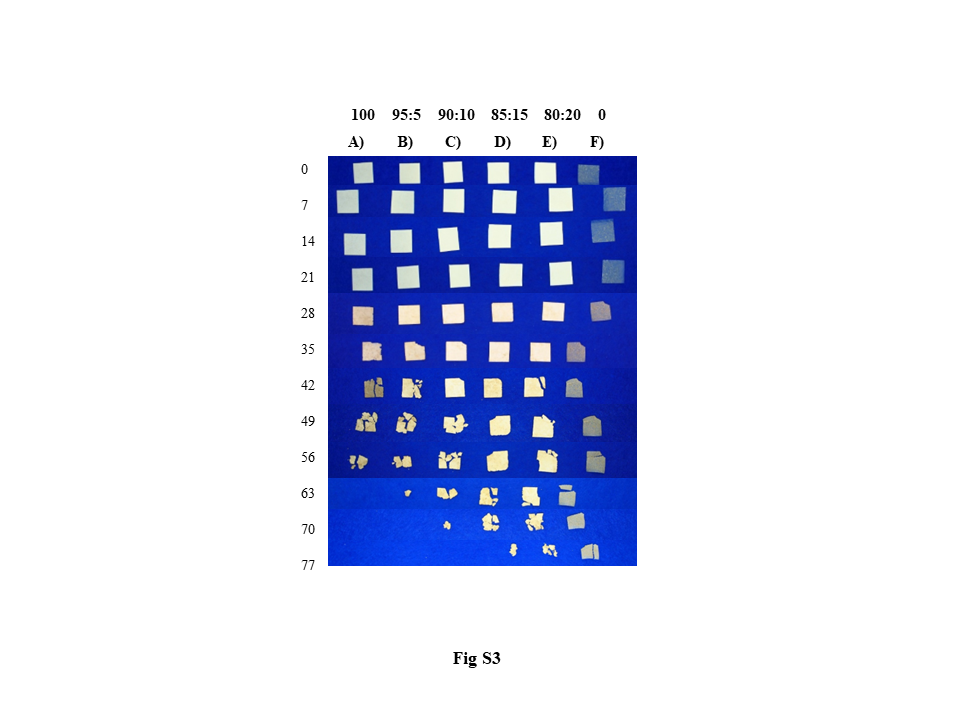

Supplement: S3 Fig — (TIF) [file pone.0292051.s003.TIF]

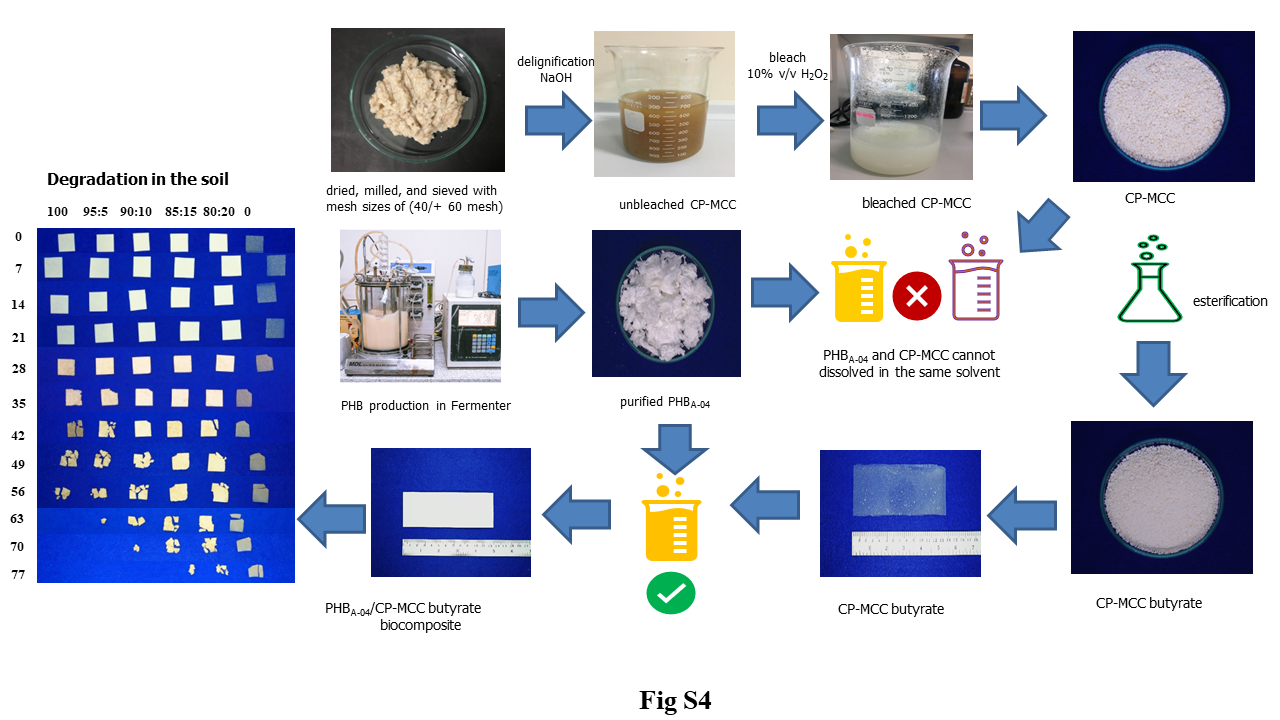

Supplement: S4 Fig — (TIF) [file pone.0292051.s004.TIF]
